# Assessing the Reliability of the ORADIII (Oral Radiology Artificial Intelligence Diagnostic - Version 3) Software Application in Rendering a Diagnosis: A Retrospective Study

**DOI:** 10.7759/cureus.104998

**Published:** 2026-03-10

**Authors:** Kavya Shankar Muttanahally, Juan Gonzalez, Gabriel P Crocker, Nagamani Narayana

**Affiliations:** 1 Oral and Maxillofacial Radiology, University of Nebraska Medical Center, Lincoln, USA; 2 Dentistry, University of Nebraska Medical Center - College of Dentistry, Lincoln, USA; 3 Oral Biology, University of Nebraska Medical Center - College of Dentistry, Lincoln, USA

**Keywords:** biopsy, cbct, jaw lesions, oradiii, oral radiologist

## Abstract

Introduction

Accurate interpretation of radiographic images is crucial for general dentists in making diagnoses and treatment decisions for their patients, yet the reliability of their diagnoses is often uncertain. This study evaluates the diagnostic efficacy of the differential diagnosis software ORADIII (Oral Radiology Artificial Intelligence Diagnostic - Version 3) in interpreting jaw lesions from cone-beam computed tomography (CBCT) scans, comparing its performance to that of an oral and maxillofacial radiologist.

Materials and methods

A total of 100 CBCT cases were selected from a clinical archive between the years 2013 and 2023, focusing on patients with jaw lesions. Among these, 85 cases were confirmed by biopsy reports. The top three differential diagnoses provided by ORADIII were analyzed, and their diagnostic accuracy was compared with that of an oral and maxillofacial radiologist who utilized clinical information and 3D-rendering software (Invivo; Anatomage Inc., Santa Clara, CA, USA). Statistical analyses were performed to evaluate the significance of differences in diagnostic accuracy between the two methods.

Results

The accuracy of ORADIII was 21% when compared to the oral radiologist’s diagnoses, which achieved an accuracy of 68% against a definitive biopsy. ORADIII accurately diagnosed dentigerous cysts in five out of six instances. However, statistical analysis revealed a significant difference at the 0.05 level, indicating that the oral radiologist's top differential diagnosis was more accurate than ORADIII's.

Conclusion

While ORADIII demonstrates potential as an adjunct tool for general dentists in diagnosing jaw lesions, it should not be relied upon as a standalone solution. The study underscores the importance of clinical expertise in achieving accurate diagnoses and highlights the need for further research to enhance ORADIII’s algorithms by incorporating additional clinical data and exploring machine-learning techniques for improved diagnostic accuracy. This study reinforces the complementary roles of technology and clinical judgment in the field of dentistry.

## Introduction

Accurate interpretation of radiographic images is fundamental to diagnosing oral pathologies and guiding clinical decisions. Advanced imaging modalities, such as cone-beam computed tomography (CBCT), have significantly enhanced clinicians' ability to visualize dental and maxillofacial structures in 3D, allowing for improved detection of pathologies, such as cysts, tumors, and bony abnormalities [1]. Despite these advancements, the interpretation of 3D images can be challenging for general dentists, as this skill often falls outside their routine scope of practice [2]. The complexity of CBCT images requires specialized knowledge, typically held by oral radiologists or pathologists, contributing to diagnostic uncertainty among general practitioners. 

Computer-assisted diagnostic systems have increasingly been explored in Oral and Maxillofacial Radiology (OMFR) to enhance diagnostic accuracy and support clinical decision-making. One such system, ORAD (Oral Radiology Diagnostic), was originally conceptualized as a probabilistic diagnostic aid based on Bayesian principles. Early foundational work by Stuart C. White introduced the ORAD program as a computer-based tool designed to analyze radiographic and clinical features of jaw lesions and to generate differential diagnoses. Building upon this concept, ORADIII represents an advanced iteration of the software, aimed at improving diagnostic performance and usability. More recently, a study by Bali et al. (2026) evaluated the diagnostic accuracy of ORADIII in the assessment of intrabony jaw lesions using panoramic radiographs [3,4], comparing its outputs with histopathological findings. Their findings highlighted the evolving role of decision-support software in oral radiology and underscored the potential of ORADIII as an adjunctive diagnostic tool within academic and research settings. 

ORADIII is a software tool originally developed for interpreting 2D panoramic images, aiding clinicians in lesion detection and standardized reporting [5]. Despite its demonstrated utility in 2D imaging, its performance on 3D CBCT scans remains largely unexplored, creating a critical gap, as general dentists increasingly rely on CBCT for diagnosis and treatment planning. This retrospective study aims to address this gap by experimentally evaluating ORADIII on CBCT datasets, comparing its top differential diagnosis with that of a trained OMFR and the definitive biopsy-confirmed diagnosis. While expert interpretation is expected to remain superior, we hypothesize that ORADIII will correctly match the definitive diagnosis in 50%-70% of cases, providing valuable preliminary insights into its potential as a diagnostic support tool and guiding future development of AI-assisted 3D imaging applications in oral radiology.

## Materials and methods

This retrospective study analyzed 100 CBCT cases selected from the Oral and Maxillofacial Pathology and Radiology clinical archive at the University of Nebraska Medical Center, Lincoln, USA, covering the period from 2013 to 2023. Approval was obtained from the Institutional Review Board (IRB) under protocol #0067-23-EX. Case selection followed a consecutive sampling approach of all CBCT scans meeting the inclusion criteria: radiographically evident bony lesions, including cysts, tumors, and other abnormalities. Exclusion criteria comprised scans without bony lesions or containing artifacts that could interfere with accurate diagnosis. The most common lesion type was cysts. Biopsy reports were available for 85 cases, serving as the gold standard for diagnostic comparison.

Two imaging tools were employed for evaluation: Invivo Dental 6 (Anatomage Inc., Santa Clara, CA, USA), a 3D imaging software enabling the Oral and Maxillofacial Radiologist to assess lesion location, size, internal structure, and effect on surrounding structures, and ORADIII, originally developed for 2D panoramic radiographs, but repurposed for CBCT analysis in this study. ORADIII required users to complete 18 structured questions covering patient demographics (age, sex, race, pain, paresthesia) and lesion characteristics (location, periphery, internal structure, density, effect on adjacent structures), generating a list of differential diagnoses.

Case evaluation protocol

Oral Radiologist Assessment

A single experienced Oral and Maxillofacial Radiologist independently evaluated all CBCT scans in multi-planar 3D view, blinded to biopsy results and ORADIII outputs. The radiologist recorded the top differential diagnosis for each case based on explicit criteria: lesion location, size, border definition, internal structure, cortical involvement, effect on adjacent teeth and structures, and associated clinical symptoms.

ORADIII Data Entry

**Figure 1:**
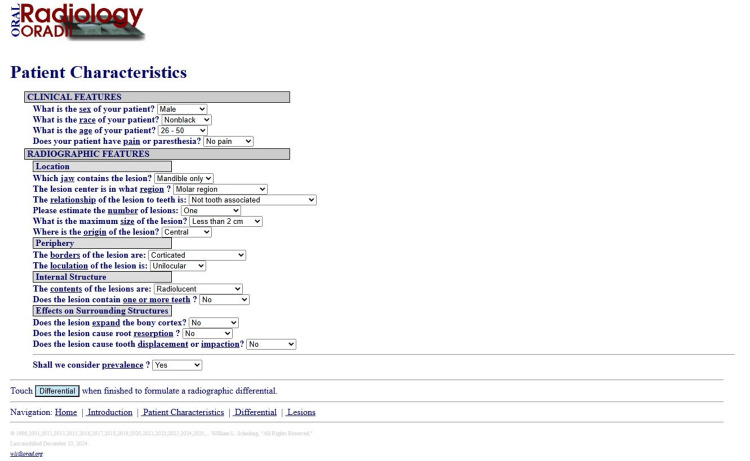
ORADIII app was employed to generate differential diagnoses

Two trained students independently entered all 18 structured responses for each case into ORADIII (Figure 1). Training included a standardized instruction session and supervised practice with sample cases to ensure consistent interpretation of imaging features. Supervision ensured adherence to protocol and consistent data entry. For each case, ORADIII generated the top three differential diagnoses, which were recorded.

Inter- and Intra-rater Reliability

To assess consistency, a subset of cases was independently entered by both students, and Cohen’s Kappa (κ) statistic quantified agreement between student entries. For the 15 cases without biopsy confirmation, Cohen’s Kappa was also used to assess agreement between ORADIII and the radiologist. Each case was entered once per student to standardize data collection.

Statistical analysis 

**Figure 2:**
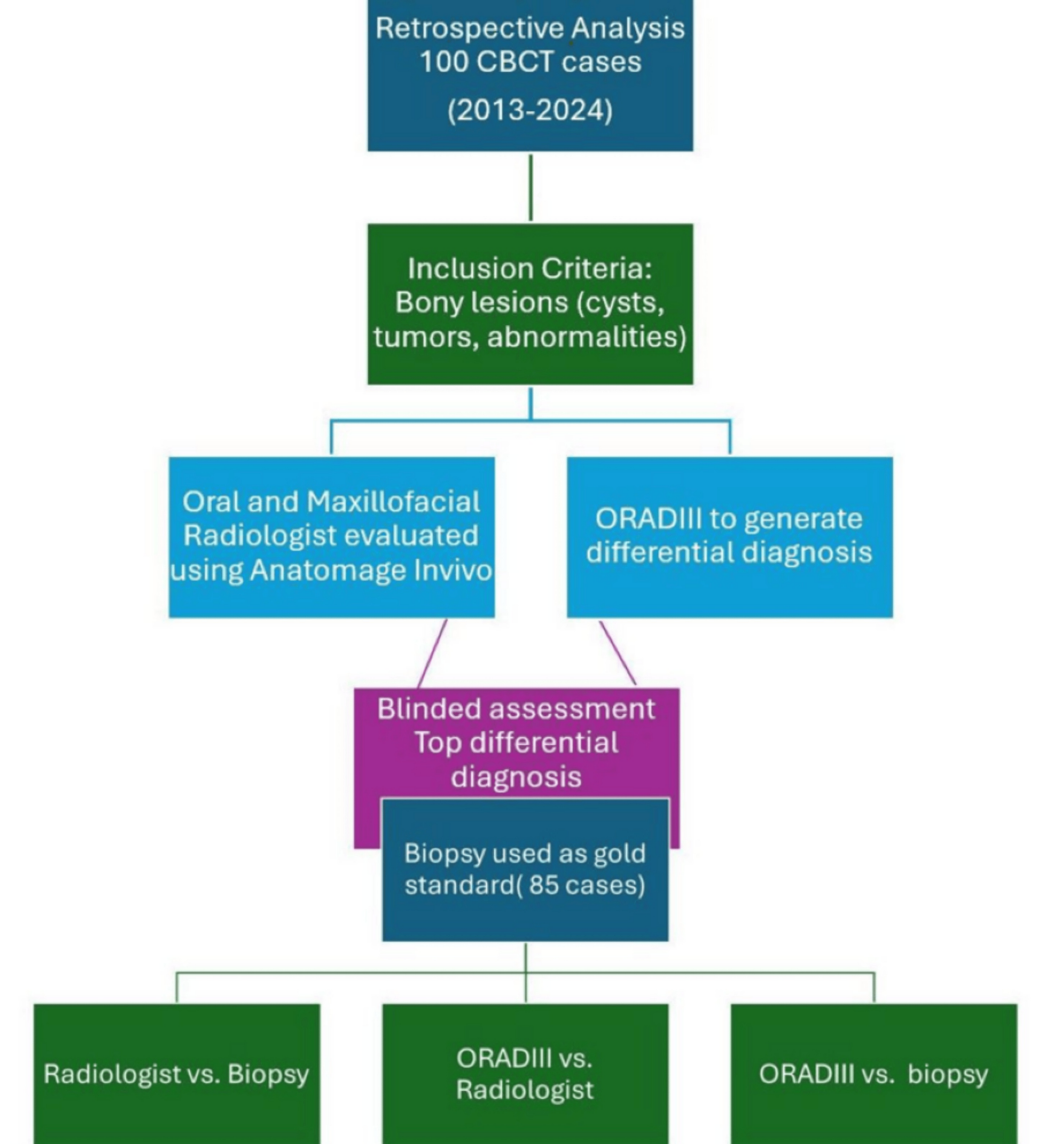
Flowchart of the materials and methods ORADIII, Oral Radiology Artificial Intelligence Diagnostic - Version 3

The primary outcome measure was diagnostic accuracy, defined as the percentage of cases in which the top differential diagnosis matched the biopsy-confirmed diagnosis (Figure 2). Diagnostic performance metrics, including sensitivity and specificity, were also calculated. Sensitivity represented the proportion of true-positive diagnoses correctly identified, whereas specificity reflected the proportion of true negatives correctly excluded.

For the 85 biopsy-confirmed cases, McNemar’s test was used to compare paired diagnostic accuracy between ORADIII and the oral and maxillofacial radiologist. A p-value < 0.05 was considered statistically significant. Chi-square (χ²) analysis was additionally performed to evaluate differences in the distribution of correct and incorrect diagnoses between the two diagnostic methods. Ninety-five percent confidence intervals (95% CI) were calculated for accuracy, sensitivity, and specificity to provide measures of statistical precision.

Agreement between ORADIII and the oral and maxillofacial radiologist for the top differential diagnosis was assessed using Cohen’s kappa (κ) coefficient. For the 85 biopsy-confirmed cases, the κ statistic quantified the level of agreement beyond chance between expert interpretation and AI-generated diagnoses. For the 15 cases without biopsy confirmation, Cohen’s κ was also calculated to assess concordance in the absence of histopathologic validation.

Inter-rater reliability for structured data entry into ORADIII by the two student evaluators was similarly assessed using Cohen’s κ to ensure consistency in data input procedures. All analyses were performed using IBM SPSS Statistics for Windows, Version 27 (Released 2019; IBM Corp., Armonk, NY, USA).

## Results

Diagnostic accuracy

Among the 85 biopsy-confirmed cases, the oral radiologist demonstrated a significantly higher level of agreement with the definitive diagnosis than ORADIII. Confidence intervals for ORADIII were wider and skewed toward lower values, reflecting greater variability in the AI system’s diagnostic performance. In contrast, the radiologist’s results exhibited narrower confidence intervals and higher accuracy, demonstrating more consistent and reliable diagnostic performance.

**Figure 3:**
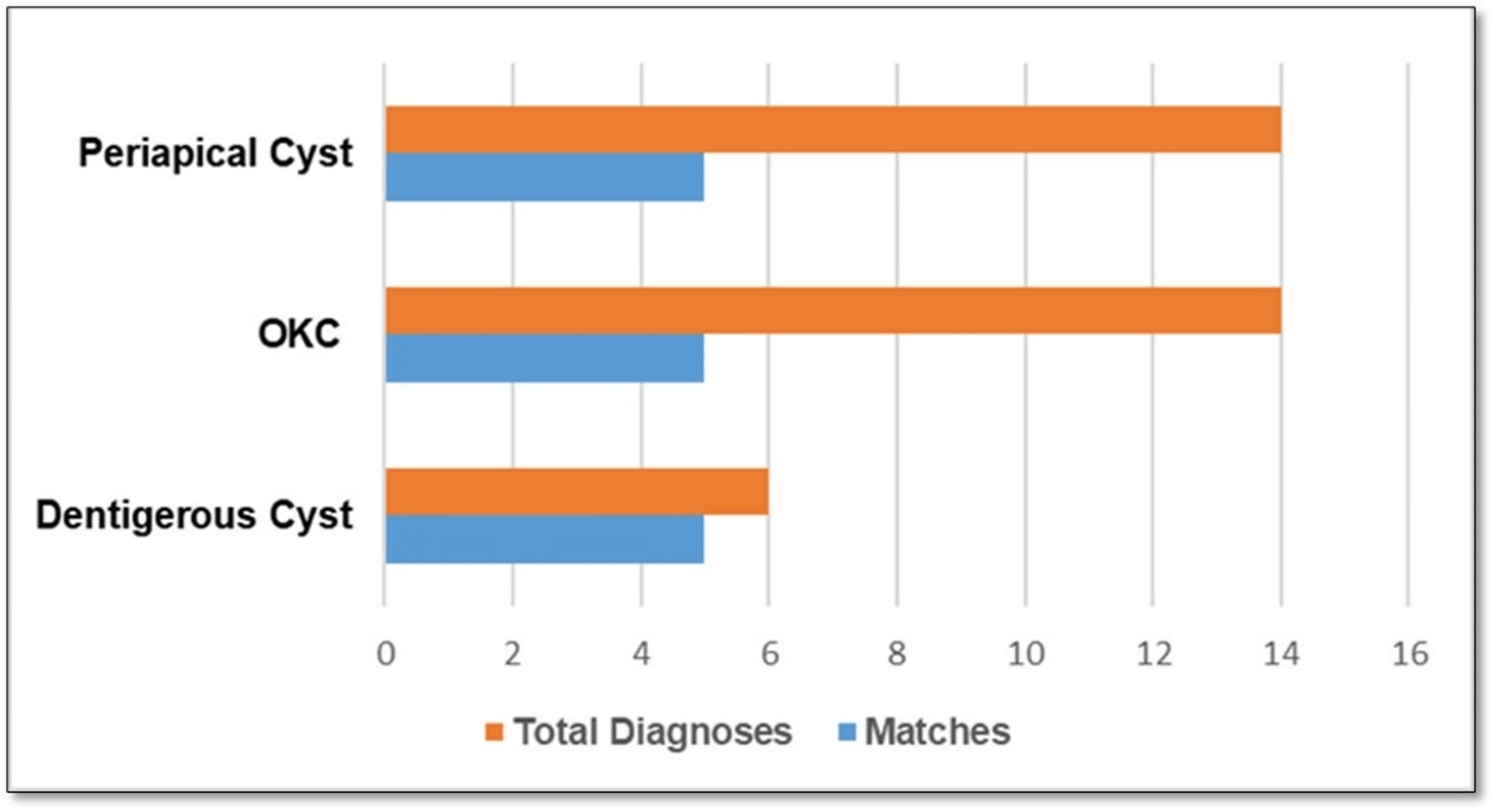
Top three differential diagnoses provided by ORADIII with their respective accuracies The orange bar denotes total diagnoses, and the blue bar denotes correct matches. OKC, Odontogenic Keratocyst; ORADIII, Oral Radiology Artificial Intelligence Diagnostic - Version 3

Figure 2 presents the top three differential diagnoses produced by ORADIII, alongside their accuracy rates for the biopsy-confirmed diagnoses. Table 1 and Figure 3 show the percentage agreement between ORADIII's top diagnosis and the definitive diagnosis, with 95% CIs. Chi-square analysis, comparing observed frequencies of correct and incorrect diagnoses, revealed a significant disparity between ORADIII and the definitive diagnosis (χ² = 34.27, p < 0.05), indicating a substantial difference in diagnostic performance. McNemar’s test similarly confirmed a statistically significant difference in diagnostic accuracy between ORADIII and the radiologist (p < 0.05).

**Table 1:** Results comparing ORADIII, the radiologist, and the definitive diagnosis ORADIII, Oral Radiology Artificial Intelligence Diagnostic - Version 3

	Matches	Total Diagnoses	Accuracy	95% CI
ORADIII vs. Radiologist	21	85	0.247	(0.155, 0.339)
ORADIII vs. Definitive Diagnosis	18	85	0.212	(0.125, 0.299)
Radiologist vs. Definitive Diagnosis	58	85	0.682	(0.583, 0.781)

The magnitude of this difference was quantified using Cramér’s V, which was calculated as 0.63, indicating a large effect size. This demonstrates that the disparity in diagnostic performance between ORADIII and expert interpretation is both statistically and clinically significant.

ORADIII demonstrated higher accuracy for dentigerous cysts, correctly identifying five of six cases (83.3%). Performance was lower for odontogenic keratocysts (OKCs), ameloblastomas, and periapical cysts, reflecting challenges in differentiating lesions with overlapping radiographic features. These findings suggest that ORADIII may be more reliable for pathologies with distinctive imaging characteristics, but struggles with complex or atypical presentations.

Inter-rater reliability for structured data entry into ORADIII demonstrated moderate agreement (κ = 0.56), indicating acceptable consistency between the two student evaluators.

Agreement between ORADIII and the oral radiologist for the top differential diagnosis in the 15 non-biopsy cases was fair (κ = 0.32), reflecting limited concordance between ORADIII diagnoses and oral radiologist interpretation when histopathologic confirmation was unavailable.

Overall, while ORADIII can generate plausible differential diagnoses in select cases, its overall diagnostic performance is significantly inferior to expert radiologic interpretation. The AI system exhibits higher variability and lower reliability, particularly in complex CBCT evaluations, highlighting the necessity for continued algorithmic refinement. Expert clinical judgment remains essential for accurate CBCT-based diagnosis.

## Discussion

**Figure 4:**
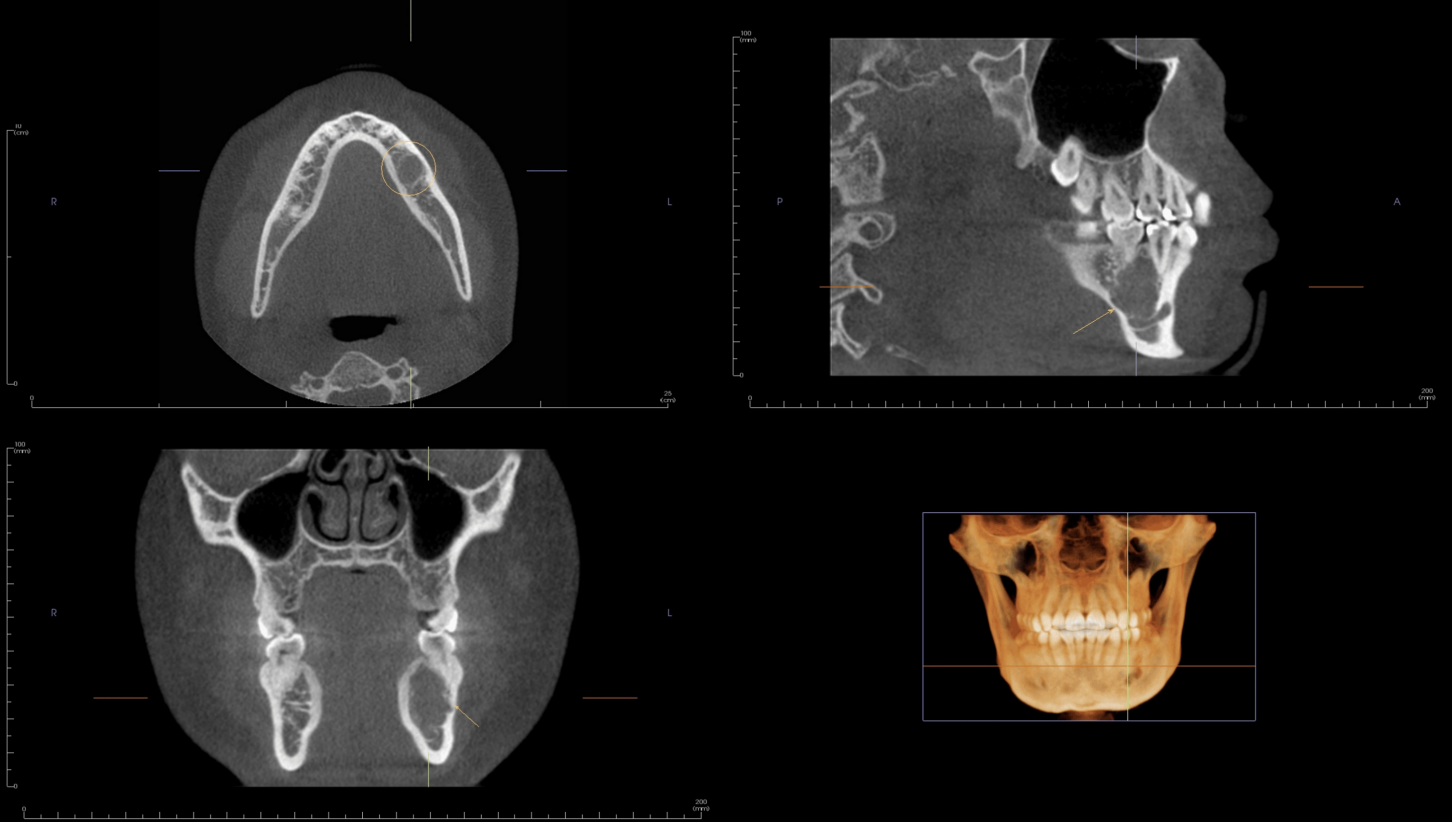
Representative CBCT scan of a jaw lesion, displayed in axial, sagittal, and coronal planes, using Invivo Dental 6 software (Anatomage Inc.) The images illustrate the lesion’s size, location, internal structure, and effect on surrounding anatomical structures. Arrows indicate the lesion boundaries, demonstrating the characteristic radiographic features used for differential diagnosis.

This study aimed to assess the efficacy of ORADIII in diagnosing hard-tissue lesions using CBCT scans, compared to an oral radiologist's assessments (Figure 4). Our findings indicated that, while ORADIII provided some accurate differential diagnoses, the oral radiologist demonstrated superior accuracy in aligning with definitive biopsy results. 

The results indicated that ORADIII's top differential diagnoses included periapical cysts, OKCs, and dentigerous cysts. Specifically, it correctly diagnosed dentigerous cysts in five out of six instances, suggesting that the software may be particularly effective in identifying this specific lesion. These findings are consistent with previous studies that highlight the potential utility of diagnostic software in assisting clinicians with complex cases, particularly in the context of odontogenic lesions [6-8]. However, the overall accuracy of ORADIII was found to be only 21% when compared to the oral radiologist and the definitive diagnosis, which is significantly lower than our hypothesis that ORADIII would correctly diagnose 50%-70% of cases. This discrepancy raises important considerations regarding the software's reliability and highlights the necessity for clinicians to utilize their clinical judgment in conjunction with technological tools [9].

The oral radiologist achieved an accuracy of 68.23% in matching the definitive diagnosis, significantly outperforming ORADIII. This finding reinforces the crucial role of clinical expertise in radiographic interpretation. Previous studies have highlighted that the nuanced understanding of an experienced clinician allows for better differentiation between similar pathologies, which automated systems may struggle to discern [10,11]. While ORADIII serves as a valuable adjunct, it should not replace the critical analysis provided by trained professionals.

Limitations of the study

Several limitations may have affected the outcomes of this study. First, only 85 of the 100 cases had biopsy reports available, limiting our ability to draw comprehensive conclusions across all cases, although these biopsy-confirmed cases provided a reliable histopathologic gold standard [12]. Second, ORADIII was originally developed for 2D panoramic imaging, and its repurposing for 3D CBCT evaluation represents an experimental application that may have reduced diagnostic accuracy. Third, although students performed data entry for ORADIII, their training and supervision were limited, and formal inter-observer reliability was assessed only for a subset of cases. Fourth, the lack of complete clinical metadata, including essential patient characteristics such as race, pain, or paresthesia, may have influenced ORADIII’s diagnostic capabilities, as these features provide critical context for accurate lesion evaluation [13,14]. Fifth, cases were selected from the available archive rather than randomized, introducing potential selection bias. Despite these limitations, the study’s strengths - including the use of 85 biopsy-confirmed cases, blinded radiologist assessment, and comparison to a histopathologic gold standard - provide valuable preliminary insights into the feasibility of using ORADIII for 3D CBCT evaluation.

Clinical applications

Despite its limitations, ORADIII demonstrates potential clinical utility as an adjunctive diagnostic tool, particularly for general dentists or practitioners with limited experience in interpreting CBCT images. For lesions with characteristic radiographic features, such as dentigerous cysts, ORADIII correctly identified 83.3% of cases, suggesting that the tool may assist in preliminary lesion identification, triaging cases, or highlighting lesions that require expert review.

By providing an automated differential diagnosis, ORADIII can serve as a decision-support tool, reducing the likelihood of overlooking obvious pathologies and supporting educational purposes in training dental students or junior clinicians. Its structured input format encourages systematic evaluation of imaging features, potentially improving consistency in preliminary lesion assessment.

Future implications and recommendations 

ORADIII has the potential to augment clinical workflow by offering rapid, standardized preliminary interpretations of CBCT scans. In busy clinical settings, such AI-assisted tools may help dentists prioritize complex cases for referral to specialists, thereby optimizing patient care. Moreover, integrating ORADIII into dental practice could contribute to digital record standardization, enabling consistent reporting of lesion characteristics, which can facilitate interdisciplinary collaboration and longitudinal patient monitoring.

Given the promising results for specific lesions, ORADIII could be integrated as a supplemental tool for general dentists, especially in complex cases where rapid decision-making is essential [15]. However, its use must be accompanied by thorough clinical evaluations and the application of additional diagnostic modalities, such as biopsy and histopathological examinations, for confirmation of lesions [7,16]. Future research should focus on improving the software's algorithms by integrating additional clinical data, enhancing its diagnostic accuracy, and validating its effectiveness across larger, more diverse populations [17,18]. The incorporation of machine-learning techniques could also be explored to further refine the diagnostic process and improve outcomes [19].

## Conclusions

While ORADIII demonstrates potential as an adjunct tool for general dentists in diagnosing jaw lesions, it should not be relied upon as a standalone solution. The study underscores the importance of clinical expertise in achieving accurate diagnoses and highlights the need for further research to enhance ORADIII’s algorithms by incorporating additional clinical data and exploring machine-learning techniques for improved diagnostic accuracy. This study reinforces the complementary roles of technology and clinical judgment in the field of dentistry. 
